# Primary atypical carcinoid of the breast: A case report and brief overview of evidence

**DOI:** 10.1186/1477-7819-9-52

**Published:** 2011-05-18

**Authors:** Iordanis Navrozoglou, Thomas Vrekoussis, Stephan Zervoudis, Mihalis Doukas, Irina Zinovieva, Andreas Fotopoulos, Minas Paschopoulos, Nicholas Plachouras, George Iatrakis, Vassilis Dousias

**Affiliations:** 1 Department of Obstetrics and Gynecology, Medical School, University of Ioannina, Greece; 2 Breast unit, Lito Maternity Hospital, Athens, Greece; 3 Department of Pathology, Medical School, University of Ioannina, Greece; 4 Department of Nuclear Medicine, Medical School, University of Ioannina, Greece

**Keywords:** atypical carcinoid, breast, neuroendocrine tumor

## Abstract

Primary atypical carcinoid of the breast is rare. Herein we present a case of atypical carcinoid of the breast treated with surgery. The management plan is commented. Moreover an overview of the current evidence is presented. All the evidence is classified as level IV (opinion-based evidence) since there is no satisfactory case series to support a certain therapeutic decision. The treatment for an atypical carcinoid of the breast is the same one offered in patients diagnosed with primary infiltrating breast cancer. A multi-centric approach is needed in order to gather enough data to confidently support a certain management plan for these patients.

## Background

Primary atypical carcinoid of the breast is considered a discrete histological entity reported in the WHO classification within the group of neuroendocrine breast tumors [1]. The exact percentage of primary breast carcinoids is unknown. Approximately 40% of the breast carcinoids are metastatic from sites well known to have neuroendocrine tissue, mainly lung, small bowel and appendix [2,3]. The rest are supposed to be primary carcinoids. However, a thorough patient investigation is needed in order to exclude an occult primary elsewhere.

Evidence regarding primary atypical breast carcinoid management is short, since this subtype of neuroendocrine breast neoplasms is considered rather rare. This does not permit large case-series to be studied and significant conclusions to be produced.

Herein, a case of primary atypical breast carcinoid is presented. Following a Pubmed search, we summarize in brief the existing evidence on the field as assistance to professionals that come up across that kind of neoplasm.

## Case presentation

The patient gave her informed consent in order for her case to be presented.

A 73-year old postmenopausal woman presented with a small nodule on the left upper medial quadrant on a routine mammography. Her medical and surgical histories were null; no family history of any malignancy was reported as well. This nodule was not detected on clinical examination, whereas no axillary lumph nodes were palpated. Breast ultrasound scanning verified the existence of a solid nodule. The patient was admitted to our department for a j-wire excisional biopsy. Routine laboratory tests and CEA, CA15-3, CA125 and CA19-9 were within normal limits.

**Figure 1:**
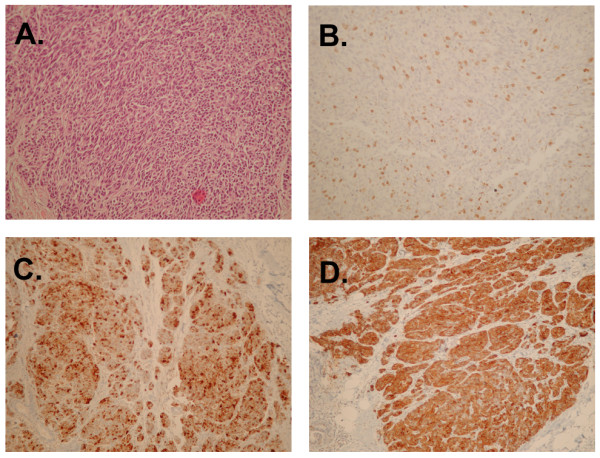
**Pathology sections (×200) of the nodule leading the diagnosis of the atypical carcinoid of the breast**: **A**. hematoxylin-eosin staining, **B**. Ki-67/MIB1 staining showing low mitotic activity, **C**. positive chromogranin and **D**. positive synaptophysin staining proving the neuroendocrine origin of the tumor.

Histology of the specimen (Figure 1) revealed a tumor measuring 1.1 cm in maximal diameter. The cut surface appeared whitish while the tumor had a nodular configuration and was hard on palpation. Microscopically the tumor was made up mostly of ovoid to round cells with variation in size, granular eosinophilic cytoplasm and nuclear pleomorhism arranged in irregular compact nests, distinct trabeculae or insular pattern of growth. Focally rosette formation was observed. Mitoses were relatively sparse. The intervening stroma was collagenised, in some areas heavily. Immunohistochemistry was positive for synaptophysin and chromogranin. There was no evidence of vascular or lymphatic invasion. The Hematoxylin-Eosin morphology assisted by the immunohistochemical expression profile confirmed the diagnosis of a carcinoid tumor with atypical features.

**Figure 2:**
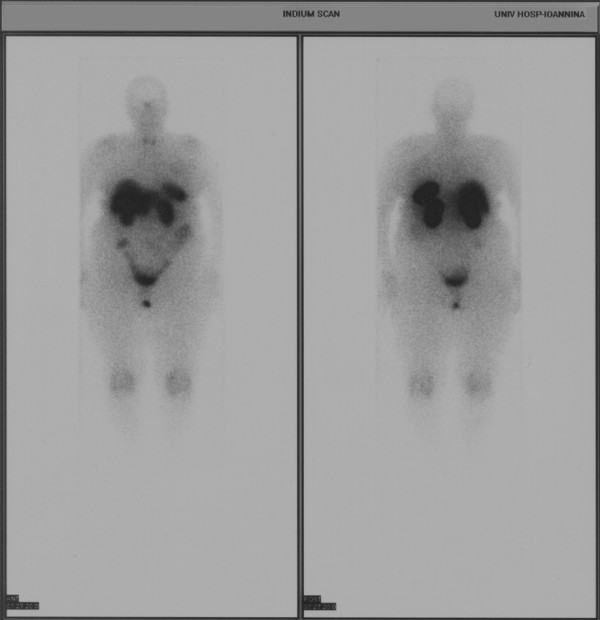
**In^111 ^- DTPA - ocreotide scintigraphy**. No evidence of increased expression of somatostatin receptors throughout the patient's body was found.

In order to exclude that being a secondary tumor stemming from a primary neuroendocrine tumor located elsewhere, a total body scan was performed after an intravenous administration of 6 mCi In^111 ^- DTPA - ocreotide. There was no evidence of increased expression of somatostatin receptor subtype 2, subtype 3 and subtype 5 throughout the patient's body (Figure 2).

Having ensured the diagnosis of a primary atypical carcinoid of the breast, the findings were discussed in the breast cancer multidisciplinary team (MDT) meeting in the view of deciding management options. A modified radical mastectomy combined with left axillary lymph node dissection (ALND), along with the decision for no further treatment in case of a negative result, was made.

Indeed the histology of the breast and of the 17 lymph nodes removed during the ALND showed no evidence of residual disease or metastatic spread and thus the patient was referred back to our unit for routine follow-up.

Four years post-operatively, the patient is in good condition with no evidence of disease.

## Case discussion

This patient was presented with a suspicious mammogram. Due to her age, the first priority was to exclude breast cancer. It was thus imperative to proceed to biopsy sampling. The decision of the j-wire biopsy was made based upon the fact that the nodule was not palpable, combined with the patient's large breast size. Primary histology result (atypical carcinoid tumor) needed further assessment to clarify whether this tumor was either primary or secondary, since primary breast carcinoids are treated mainly with a surgical approach; if this lesion was a metastatic carcinoid, nothing further was to be done breast-wise, since metastatic breast carcinoids are simply removed (lumpectomy) as part of the management plan required for the primary carcinoid treatment. In^111 ^- DTPA - ocreotide scintigraphy is considered an accepted method to verify the existence or not of a carcinoid tumor throughout the body [4]. The MDT decision for modified radical mastectomy is within the treatment options applied so far in primary atypical carcinoid tumours. However based on the slow growth rate of such neoplasms, it could be argued that such an option was an over-treatment. In our case the option of ALND alone, combined or not with breast radiotherapy was discussed with the patient prior to MDT discussion. She opted for more aggressive surgical treatment in the view of avoiding radiotherapy.

## Brief overview of evidence

Primary carcinoid tumors of the breast are considered rare, representing less than 1% of the breast tumors [3]. Atypical carcinoid breast tumors are expected to exceed 100 reported cases world-wide [5]. Most of the primary breast carcinoids are found in patients older than 65 years [6].

Primary carcinoid tumors of the breast are neuroendocrine tumors [1]. In accordance to carcinoids developed in other sites, they are classified as "typical" or "atypical" depending on the degree of cellular differentiation recognized on the specimen. The first category is characterized by neuroendocrine differentiation with classical histological architecture of cellular neuroendocrine clusters and sparse mitoses [7]. The second category refers to poorly differentiated neuroendocrine tissue with an increased mitotic index [7].

Histology reveals a uniform population of eosinophilic cells with round nuclei characterized by "salt-and-pepper" chromatin [3]. The cells seem to be organized either in nests or in strands mimicking either ductal or lobular carcinomas. Immunohistochemistry usually reveals positive reactivity for neuron specific enolase (NSE), synaptophysin and chromogranin.

The diagnostic approach of breast carcinoid tumors is the same one applied to any breast lesion. Clinical examination may or may not reveal a palpable nodule. Mammography is the imaging method of choice. Mammographic findings are those of neuroendocrine breast tumors, including dense nodules featured by well-circumscribed or irregular margins, with or without microcalcifications [8,9]. The heterogeneity of the mammographic findings can introduce difficulties in discriminating a carcinoid tumor from breast cancer. In case of clinical suspicion, however, caution is needed in order to avoid provoking a carcinoid crisis as the result of breast compression during mammography [10]. Tumor sampling by fine needle aspiration and cytology cannot always exclude invasive carcinoma [11]. In that case immunocytochemistry can assist in the diagnosis [11]. However the risk of misdiagnosing a carcinoid tumor, an incident that may lead the clinician to more aggressive management, has supported the adoption of either core [12] or excisional biopsy as the gold standard for diagnosing such tumors.

Treatment is primarily surgery ranging from breast conserving surgery or mastectomy followed by axillary lymph-node dissection to modified radical mastectomy [3,6,13]. Radiotherapy is a controversial therapeutic option in primary breast carcinoids [3,7]. However, radiotherapy can be justified if we admit that primary carcinoids are treated in terms similar to primary breast cancer. No report has been made so far, regarding either adjuvant chemotherapy or somatostatin analogues in primary breast carcinoid tumors.

To date there is no standardized treatment regarding carcinoid tumors of the breast. In most cases carcinoid tumors are approached as infiltrative carcinomas of the breast. Additionally, the reported follow-up intervals are rather short (less than 5 years), thus it is almost impossible to extract conclusions about treatment efficacy and prognosis.

## Conclusions

Primary atypical carcinoid of the breast is a rare entity. Its diagnosis requires exclusion of this being a metastasis originating from a primary carcinoid located elsewhere in the body. Treatment is mainly surgical. A multi-centric approach is needed to organize and evaluate a well-powered case series to extract significant conclusions regarding patient management and prognosis.

## Consent

Written informed consent was obtained from the patient for publication of this Case report and any accompanying images. A copy of the written consent is available for review by the Editor-in-Chief of this journal.

## List of abbreviations

MDT: Multidisciplinary Team; ALND: Axillary Lymph Node Dissection.

## Competing interests

The authors declare that they have no competing interests.

## Authors' contributions

IN and TV performed the literature search, wrote the main body of the text and revised the manuscript. SZ and GI participated in drafting the manuscript. IZ and MD evaluated histology and performed the IHC. AF interpreted the scans and participated in manuscript drafting. NP, MP and VD critically revised the manuscript. All authors have read and approved the final version of the manuscript.
